# A systems-based mathematical modelling framework for investigating the effect of drugs on solid tumours

**DOI:** 10.1186/1742-4682-8-45

**Published:** 2011-12-07

**Authors:** Cong Liu, J Krishnan, Xiao Yun Xu

**Affiliations:** 1 Chemical Engineering and Chemical Technology, Imperial College London, South Kensington, London SW7 2AZ, UK; 2 Centre for Process Systems Engineering and Institute for Systems and Synthetic Biology, Imperial College London, South Kensington, London SW7 2AZ, UK

**Keywords:** Solid tumour, drug effect, transport, intracellular signalling, systems approach, modelling framework, bottom-up approach.

## Abstract

**Background:**

Elucidating the effects of drugs on solid tumours is a highly challenging multi-level problem, since this involves many complexities associated with transport and cellular response, which in turn is characterized by highly non-linear chemical signal transduction. Appropriate systems frameworks are needed to seriously address the sources of these complexities, especially from the cellular side.

**Results:**

We develop a skeletal modelling framework incorporating interstitial drug transport, intracellular signal processing and cell population descriptions. The descriptions aim to appropriately capture the nature of information flow. The model is deliberately formulated to start with simple intracellular descriptions so that additional features can be incorporated in a modular fashion. Two kinds of intracellular signalling modules which describe the drug effect were considered, one a monostable switch and the other a bistable switch. Analysis of our model revealed how different drug stimuli can lead to cell killing in the tumour. Interestingly both modules considered exhibited similar trends. The effects of important parameters were also studied.

**Conclusions:**

We have created a predictive systems platform integrating drug transport and cellular response which can be systematically augmented to include additional layers of cellular complexity. Our results indicate that intracellular signalling models which are qualitatively different can give rise to similar behaviour to simple (and typical) stimuli, and that validating intracellular descriptions must be performed with care by considering a variety of drug stimuli.

## Background

The need to systematically understand the complex aspects of solid tumours is evident when one considers the potentially fatal consequences which are associated with solid tumours growing unchecked. Solid tumours are a highly complex mini-universe in themselves. They are typically fed by a complex vascular network which provides blood and nutrients. This vascular network is itself more complex and irregular than vascular networks in normal tissues. The interstitium (the region of the tumour other than the vascular network) contains the tumour cells as well as the extracellular matrix. It is worth pointing out that even such a picture masks important events that occur at different time scales. For instance, a growing tumour which is not vascularized, secretes chemicals which eventually lead to its vascularization by the process of tumour-induced angiogenesis.

The complexity of the tumour environment becomes even more relevant when one attempts to evaluate systematically the effects of anti-cancer drug on tumours. Different drugs such as doxorubicin and paclitaxel have been used (and delivered in different forms) with the aim of effectively destroying tumour cells. These drugs are typically injected into the blood stream and enter the interstitium through the capillary wall. After entering the interstitium they diffuse in the interstitial space, where they may also bind to albumin or other proteins [1]. The unbound drug may be taken up by tumour cells, upon which they can act. Clearly, a number of complexities must be considered when one attempts to develop a mechanistic understanding of the effect of drug on solid tumours. These include the complex microvasculature as well as the complex structure of the interstitium [1]. Moreover, it is necessary to understand the highly non-linear nature of the cellular response in tumours and how this is affected by the tumour microenvironment [2], including both chemical and biophysical aspects.

Several attempts have been made to model mathematically the effect of drug on solid tumours [3]. These include compartmental models describing the tumour as single or discrete compartments [4,5], transport models focusing primarily on blood flow and drug diffusion in tumours [6,7] and pharmacokinetic and pharmacodynamic models including varying levels of description of the intracellular response. Recent computational work has begun to focus on combining interstitial transport with drug uptake by cells [8]. While all these models provide varying levels of insights, there are no models that offer a transparent systems level description of the constituent elements, with a dynamical systems basis for the description of the cellular signalling.

In this paper we take the first steps towards developing an integrative modelling framework which combines blood flow and interstitial transport, while also systematically accounting for the complexity of the relevant signal transduction in tumour cells. In the last decade, considerable amounts of interest and research activity have focussed on unravelling the intracellular and intercellular signal transduction, under the broad umbrella of Systems Biology. The approach of systems biology is especially relevant in the current context as many aspects of the cellular response to drugs in tumours are not systematically understood, and these (along with other aspects of cellular communication and interaction) may play important and unexpected roles in the actual cellular response. A better understanding of these complex interactions could also pave the way for refining treatment options. Indeed, an increasing awareness of the need for a "systems pharmacology" approach is being advocated [9], which will require a mechanistic description of tumour response, as well as intracellular biochemical interactions, with additional elements such as variability or patient specific information built into this.

Therefore, the aim of this study is to formulate a spatially distributed model to address the effects of anti-cancer drugs on tumour tissues, with a particular focus on the complexities of cellular response. This is achieved by integrating interstitial transport and cellular response, the latter presenting a considerable challenge given the complexity of cellular signalling and the high degree of nonlinearity in cellular signal processing. A unique feature of our approach is to account for the important aspects of intracellular response in a manner which is transparent and modular, so that the models can be systematically refined and augmented. This is very important as it offers the opportunity for further expansion, including incorporation of the powerful tools of systems biology in the proposed modelling framework. Our framework may thus be viewed as one which bridges the gap between pharmacodynamic type modelling and detailed mechanistic systems biology modelling, while being firmly rooted in dynamical systems approaches. Using the models presented here, analyses can then focus on understanding the effects of potentially important contributing factors, such as the nature of the intracellular signalling, the microenvironment and how this differs from tumour to tumour, the effect of intrinsic stochasticity as well as cellular variability.

## Methods

### Modelling strategy

It is recognized that the problem under consideration features a genuinely complex system, and hence any model building attempt must make choices regarding what features are central. In the work presented here, some explicit descriptions of the cellular processes are included, expanding on the modelling description of Eikenberry [8]. However, a conundrum we must face is that despite a concerted and focussed effort leading to modelling advances, many mechanistic gaps in understanding the apoptosis network (the key step in cell killing) remain. In addition, mechanistic elucidation of the role of signalling in apoptosis was performed in normal rather than tumour cells. Therefore, it is important to incorporate cellular effects at an appropriate level, so that the key features of the cellular and tissue dynamics are included without having to wait for all the relevant cellular networks to be fully understood. This informs our modelling approach: we use a combination of coarse grained descriptions of the cellular signalling dynamics, but also examine these alongside prototypical detailed models (discussed briefly) to see if there is any essential difference between the two.

The modelling framework presented here is intended to be used as a skeletal systems platform for gradual expansion to include further cellular/flow complexity. Thus data fitting is not attempted at this stage. Our model descriptions for cells are continuum based. This is to allow the additional tools available for continuum type analysis to be utilised as further complexity is added in the future (note the comments in Hinkelmann *et al*. [10] regarding discrete models).

### Model assumptions

**Figure 1:**
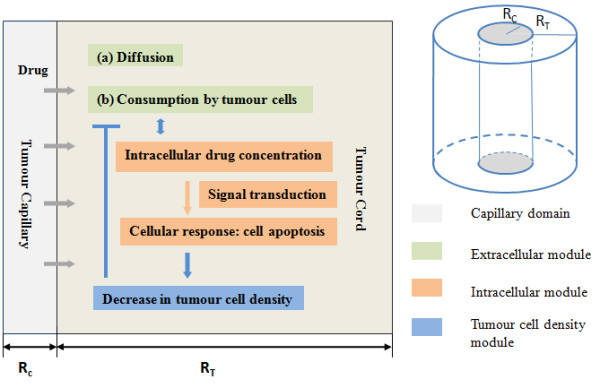
**Diagrammatical representation of the tumour cord model (right) and the levels of description incorporated in the modelling framework (left)**.

The model is based on the description of drug distribution and cells in a fixed cylindrical tumour cord (Figure 1) where the cells are assumed to be stationary and alive initially. In this setting, the tumour interstitium is represented as a cylindrical annulus, with the inner radius (R_C_) corresponding to the capillary radius and the outer radius (R_T_) for the tumour cord. To simulate drug distribution in the interstitium, it is assumed that drug is injected directly, either at the entry point of the tumour or at some other location in the body, each resulting in a specific drug concentration at the capillary wall, which is the inner boundary of the tumour cord. Different forms of drug infusion and input drug concentrations are examined.

The main assumptions of the model are (i) Cells are initially alive and present at uniform density in the tumour-cord (ii) There is minimal cell movement in the tumour cord (iii) Prior to injection of the drug, conditions are uniform inside the tumour cord (iv) Cellular variability, stochasticity, and effects of cell-to-cell interaction (mechanical and chemical) and cell-cycle are ignored.

### Extracellular drug transport

The extracellular drug distribution in the tumour cord is determined by a balance of diffusion, cellular uptake, and cellular release (pumping out) into the interstitium and in addition the binding to, or unbinding from proteins such as albumin, similar to [8]. The convective effects in the interstitium are neglected, as is customary, and spatial variation of the drug in the tumour cord is assumed to occur in the radial direction only. Incorporating all these factors, the dynamics of the extracellular concentration is governed by

(1) $$\frac{\partial E}{\partial t}=k_{d}\nabla^{2}E+c_{t}\left(\frac{V_{2}I}{k_{2}+I}-\frac{V_{1}E}{k_{1}+E}\right)-k_{3}E+k_{4}B$$

where E refers to the extracellular drug concentration (a function of radial position and time), k_d _is the diffusion coefficient of the drug in the interstitium while c_t _is the cell density. The two terms in the equation which involve the cell density describe the pumping out of intracellular drug concentration (denoted by I) and the cellular uptake of the extracellular drug concentration. Both these terms are described by sigmoidal saturating functions. The last two terms refer to the binding to and unbinding from proteins such as albumin, present in the interstitium. B denotes the concentration of bound extracellular drug.

The equation for the dynamics of the bound drug is given by

(2) $$\frac{\partial B}{\partial t}=k_{db}\nabla^{2}B+k_{3}E-k_{4}B$$

where k_db _is the diffusion coefficient of the bound drug.

The non-flux condition is applied for both free and bound drug concentrations at the outer boundary, whereas at the inner boundary the well-known Kedem-Katchalsky equation [11] is specified,

(3) $$F_{E}=K\left(S-E\left(r_{c}\right)\right)+K_{1}$$

which prescribes the flux F_E _at the capillary wall as the sum of two terms. The first term is proportional to the difference between the drug concentration in the capillary S, and the extracellular concentration at the edge of the interstitium (E(r_c_)) and depends on the permeability of the capillary wall (K). The second term describes a convective flux through the capillary wall, where the transmural velocity is described by Starling's Law.

Similarly an equation describing the intracellular concentration of drug can be written, which depends on the pumping in and pumping out of drugs, similar to [8] (though the possible effects of drug sequestration will also be examined later).

(4) $$\frac{\partial I}{\partial t}=\frac{V_{1}E}{K_{1}+E}-\frac{V_{2}I}{K_{2}+I}$$

### Tumour cell density

The equation governing the tumour cell density describes a balance between growth and death of cells. Although a more comprehensive description of birth and death of cells can be achieved in population balance formalism, this approach is computationally highly demanding and will be difficult to handle as additional cellular complexity is added. Here cell densities are described by ordinary differential equations. The ODEs are logistic equations [12], and naturally describe the growth and death, with a balance leading to a steady state. These equations naturally incorporate saturating growth (or equivalently non-linear death rates) leading to a finite steady state. The equation governing the cell density is given by

(5) $$\frac{\partial c_{t}}{\partial t}=ac_{t}-bc_{t}^{2}$$

Note that the logistic equation admits two steady states c_t _= 0 and c_t _= a/b, as long as a > 0. The latter steady state is the stable steady state. However, when a < 0 the only biologically relevant state is the zero steady state.

Eqn (5) can arise in two somewhat different ways giving the same essential result: linear growth rate (a_1_c_t_) and a quadratic death rate (a_2_c_t _+ bc_t_^2^). The net growth rate is given by (a_1 _- a_2_)c_t _- bc_t_^2^, which is exactly of the above form if a_1 _> a_2_. This description is adopted here. A second way in which a very similar equation results is if the growth rate is a saturating function of cell density and equal to Ac_t_/(A_1 _+ c_t_), where A is the maximal growth rate, and the death rate is given by Bc_t_. Here A, A_1 _and B are all constants. Writing out the equation for c_t _in terms of growth and death gives

(6) $$\frac{\partial c_{t}}{\partial t}=\left[\left(A-A_{1}B\right)c_{t}-Bc_{t}^{2}\right]/\left(A_{1}+c_{t}\right)$$

It is clear that this equation has a very similar form to Eqn (5) (except for the denominator) and in fact the qualitative property of the steady states (the dynamical systems aspect of interest) is essentially identical to that described above. Thus the logistic equation is adopted here, as it includes growth and death with saturating effects. It can be noted that if a_1 _< a_2 _Eqn (6) has only one physical steady state which is c_t _= 0.

### Intracellular signalling

The modelling of intracellular signalling processes forms a substantial core of systems biology. Given the complexity of signalling, it is a non-trivial issue as to what the appropriate level of description should be, more so since there are many missing biological details in the signalling and many parameters unknown. At the same time there is an urgent need to start accounting for known and nontrivial aspects of the signalling, so that a predictive platform can be built for examining the effects of different cellular (intracellular or even intercellular) features. An appropriate degree of complexity is necessary for systematic evaluation and analysis of data.

As described earlier, the strategy adopted here is to start with simpler descriptions, and increase the degree of complexity gradually. In doing so, it is also important to represent the nature of the information flow correctly, and ensure that the qualitatively important features of detailed models must be accounted for. Sample detailed models in the literature are used to check the validity of the simplified models, especially for the type of drug signals encountered by cells.

In modelling the intracellular processes involving drugs, the main process of interest is drug induced apoptosis. Here, there are two key features that any model, detailed or simplified, must reflect. Firstly there must be some threshold effect present, and secondly and even more importantly, the "switch" to apoptosis must be realized in an irreversible way. Based on the systems biology literature, two types of switches are usually observed -monostable and bistable switches. Both switches are widely observed in cellular signalling generally but have very different signal transduction properties; monostable switches are completely reversible, while bistable switches can exhibit irreversibility and this has led them to be used in the modelling of irreversible processes.

Bistable processes have indeed been used in modelling irreversible cell fate decisions leading to apoptosis [13,14]. Such modelling uses positive feedback in the caspase network (which is known to biologically exist) as a source of generating bistability. In the case of apoptosis in particular, it is not at all obvious that the irreversible fate (cell death) is necessarily or even reasonably (at the cell fate decision level) reflected as a steady state: it is very possible that an irreversible decision can lead to the triggering of critical cellular events from which there is no turning back. Thus the irreversibility could be reflected as a simple irreversible reaction, which is triggered only under very special circumstances [15]. Noting this, two models are employed here: (1) a sequential interconnection of a monostable switch and a downstream irreversible reaction effect, and (2) a bistable switch. The latter has self-contained threshold behaviour and irreversibility. It is noted that a combination of a bistable switch with an irreversible downstream reaction could also occur, with the bistable switch providing the threshold, and the downstream reaction providing the irreversible nature of the response (discussed later).

The equation of the monostable switch is given below. The upstream signal which is the input to this module is the intracellular drug concentration, and the model employs a Hill-type non-linearity. The range of response is normalized.

(7) $$\frac{dR}{dt}=k\left(\frac{I^{n}}{k_{h}+I^{n}}-R\right)$$

where n denotes the Hill coefficient, k_h _an associated constant in the Hill term, and k is a parameter representing the time scale of the response. The above equation simply depicts the element R responding in a non-linear fashion to the input I. When the Hill exponent n is increased the steady state input-output response approaches a switch-like function. The above model is one concrete representation of a (monostable) switch like function: for each input value, there is only one steady state. We mention that monostable switch responses could also be realized through other means in signalling, for instance opposing enzymes acting at saturation, or the combination of stage-wise effects in a signalling cascade. However for our purposes all these models exhibit very similar input-output characteristics.

The response drives a second reaction which describes the activation of a species R_1 _from its inactive form.

(8) $$\frac{dR_{1}}{dt}=k_{f}R\left(1-R_{1}\right)-k_{r}R_{1}$$

Note that in the above, under basal conditions R_1 _is very small and can only reach appreciable levels when the R level is high enough (for a substantial period of time). In our model cell death is triggered if R_1 _reaches a particular threshold R_o_, and this is reflected at the population level in an irreversible manner. Note that for R_1 _to be above the threshold, the intracellular drug concentration must drive the upstream signal (R) above the threshold, and keep it there for a sufficient period of time.

In the above model, R represents a typical downstream intermediate element in the signalling cascade, while R_1 _represents a signal responsible for directly triggering apoptosis (and may be regarded as the output of the cascade).

It is worth also pointing out that for both the monostable and bistable modules, our signalling models are essentially minimal, but include a key intermediate step connecting input to response. This is done so that the qualitative dynamics is correctly captured, and also so that additional complexity or other factors can be appropriately incorporated.

The second model adopted is a sample bistable model which arises from a positive feedback. While many similar models with minor variations have been examined and simulated, (some employ Hill-type cooperative effects and others do not), the model chosen here has saturated degradation with positive feedback [16]. The upstream signal plays a role in catalyzing this positive feedback pathway (either independently or together with other existing enzymes). This module describes the concentration of the active form of a protein R which involves the activation and deactivation reactions, as well as positive feedback from the active form of the protein in further activating inactive protein. Here again, the total concentration of the protein is normalized to be 1. The governing equations are

(9) $$\frac{dR}{dt}=\frac{V_{f}\left(1-R\right)}{K_{m1}+\left(1-R\right)}+\left(p+qI\right)k_{fb}R\left(1-R\right)-\frac{V_{r}R}{K_{m2}+R}$$

where K_m1 _and K_m2 _are Michaelis Menten parameters. k_fb _is a kinetic parameter which parametrizes the feedback strength. Note that the upstream signal I enzymatically acts to trigger the switch. The constants p and q serve to set the basal level and dynamic range of the module. The factor p describes the feedback present under basal conditions in the network (i.e. even in the absence of the stimulus I): even in the absence of the stimulus, the feedback results in a bistable circuit. This allows for the fact that even after the removal of a stimulus, an irreversible change in output could occur, and is exactly the way in which bistable signal processing (in the caspase network) leading to irreversible decision making in apoptosis is invoked [13].

The bistable switch has the feature that when the signal I crosses a threshold value, a sharp increase in the steady state of R is observed (see Appendix for more details). It should be noted that other variants of bistable modules examined all possess essentially similar features and input-output characteristics. In all these cases an upstream signal leads to the triggering of the bistable switch. While the equations of different modules are different in details, the key dynamical systems aspect of interest is in fact very similar (and manifests itself in very similar qualitative behaviour of these modules to signals of the kind encountered here). Here the output of this reaction drives the same activation of R_1 _just as before.

Cell death is triggered by a threshold of R_1 _being crossed. Note that the irreversibility could arise, simply by the threshold (in the bistable module) being crossed for enough time by the upstream signal. Thus even in the case where the upstream signal is transient, a different steady state of the bistable module can be attained, signifying an irreversible response. In contrast to the monostable switch, the effect of the crossing of a threshold in R_1 _on cell population density is modelled in a reversible manner. Thus in this case the irreversibility arises in the bistable switch itself: if it is permanently switched on (for instance even in a transient signal), then this will be reflected as cell death at that location in the population level.

Suitable representative parameters are employed for both switch modules. Although detailed quantitative comparison between the two models is not attempted (given their different dynamics), we seek to examine if the qualitative differences in these models are reflected at the population level. Therefore the parameter values for the two models are chosen in such a way that the threshold (of the intracellular drug concentration) for switching on is the same in both, and that they have comparable time scales. The latter is achieved by ensuring that the time to killing is the same for a reference signal value in both models.

The only remaining point is how to represent cell death at the population level. Noting that the cell population density is described in a continuous, rather than discrete form, the triggering of the intracellular threshold is incorporated by a sharply (or completely) reduced growth rate at the population level. From the dynamics of the logistic model, it is clear that if the growth rate becomes sufficiently low (i.e. a_1 _< a_2_), eventually all the cells will perish. The same effect could also be achieved by increasing the death rate by adding an extra linear term. Both cases gave essentially similar results. Importantly, in the computation, this threshold effect is implemented in a unidirectional (irreversible) way in the monostable model but in a reversible way in the bistable model.

All the above equations are written in dimensionless form. The non-dimensionalization has been performed as follows: the spatial co-ordinate is non-dimensionalized by the outer radius, time by a typical diffusion time scale; the intra and extra cellular concentrations by reference concentrations which have representative values (0.001 μg/mm^3 ^for extracellular, 1 ng/10^5 ^cells for intracellular), and the tumour cell density is non-dimensionalized by a typical value (10^6 ^cells/mm^3^). Likewise the input signal (denoted by S) is non-dimensionalized by the same representative factor (0.001 μg/mm^3^). Model parameters are determined as follows: based on non-dimensionalization, the size of the domain and time scales, parameters are derived either directly or indirectly from the literature, for the drug doxorubicin. The tumour growth parameters are chosen to reflect both the range of steady state, as well as the appropriate time scale. In the intracellular dynamics, the threshold to activate cell death and the time scale of the activating pathways are of special interest, and the variations of these parameters are examined here. Essential model parameters used in the present study are summarised in Tables 1 and 2. We emphasize that many of our essential conclusions are not strongly dependent on the choice of parameters.

**Table 1:** Parameters and values used in extracellular drug transport and uptake.

Parameter	Symbol	Value	Reference
Free DOX diff. coefficient	k_d_	0.568 mm[2]/hr	[2]
Bound DOX diff. coefficient	k_db_	0.032 mm[2]/hr	[2]
Rate of transmembrane transport	V_1_, V_2_ (V_1 _= V_2_)	0.28 ng/(10^5 ^cells)/min	[4]
Michaelis constant for transmembrane transport	k_1_	0.219 μg/ml	[4]
Michaelis constant for transmembrane transport	k_2_	1.37 ng/(10^5 ^cells)	[4]
Free DOX-albumin binding rate	k_3_	3000 hr-^1^	[2]
DOX-albumin dissociation rate	k_4_	1000 hr-^1^	[2]
Initial tumour cell density	C_t,0_	10^6 ^cells/mm^3^	[2]
Tumour cell growth rate	a_1_	0.5 day^-1^	Estimated
Saturation constant in logistic equation	b	0.02592 mm^3^/(10^5 ^cells)/day	Estimated
Tumour cell natural decay rate	a_2_	0.24 day^-1^	Estimated
Tumour capillary radius	R_C_	10 μm	[2]
Tumour cord radius	R_T_	120 μm	[2]

Doxorubicin (DOX) is chosen as the anti-cancer drug for parameterization purpose.

**Table 2:** Parameters and values used in intracellular signal transduction modules.

Parameter	Symbol	Value	Reference
Bistable switch			
Michaelis Menten constants	V_f_	27 hr^-1^	[6]
Michaelis Menten constants	V_r_	0.459 hr^-1^	[6]
Michaelis Menten constants	K_m1_	100	[6]
Michaelis Menten constants	K_m2_	0.01	[6]
Kinetic parameter mediating feedback strength	k_fb_	2.927 hr^-1^	[6]
Basal parameter in the bistable switch	p	0.7	Estimated
Parameter mediating input regulation in the bistable switch	q	0.3(ng/(10^5 ^cells))^-1^	Estimated
Monostable switch			
Kinetic parameter reflecting the time scale of the response	k	0.432 hr^-1^	Estimated
Associated constant	k_h_	1 ng/(10^5 ^cells)	Estimated
Hill coefficient	n	10	Estimated
R_1 _protein activation rate	k_f_	3.6 hr^-1^	Estimated
R_1 _protein degradation rate	k_r_	0.144 hr^-1^	Estimated
R_1 _Threshold for apoptosis switch	R_1_,_th_	0.9	Estimated

Model simulations were performed in MATLAB by suitably discretizing the diffusion in the radial direction in a standard way using finite differences [17], and solving the resulting set of ODEs using the solver ode15 s. The effect of the discretization was checked by doubling the mesh points. Further the simulation results in a few sample cases were also checked with simulations performed in COMSOL.

## Results

We present the results of the analysis of our model by considering and contrasting the results for multiple variants of the intracellular signal transduction considered. In the first case, constant levels of drug input at the capillary wall are examined, which provides important information about how the drug signal information arrives, and is processed by the tumour cells. Following this, more complex forms of drug input are analysed, specifically in the form of a single bolus, followed by multiple bolus injections. The effects of other important auxiliary parameters are also examined. A related discussion with some analytical insight is presented in the Appendix.

**Figure 2:**
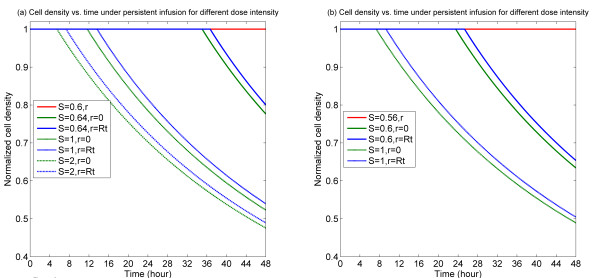
**Temporal profiles of cell density at specific spatial locations for persistent infusion for different dose intensities**. (a) Bistable switch case, (b) Monostable switch case. It is noted in the current and following figures that multiple plots might overlay one another at cell density equal to 1. In each of the plots, the red line indicates the level of cell density (spatially uniform) for a below threshold dose, showing that no killing results in this case.

We consider direct injections of drug, or liposomal release, at the entry to the tumour cord. Drug input is represented by the drug concentration in the capillary, through the Kedem-Katchalsky boundary condition. For constant input drug concentrations, results shown in Figure 2 suggest that when the drug concentration is relatively low, no tumour cells are destroyed. Thus even with a constant stimulus of drug, the system evolves to a steady state where the cells essentially remain intact. On the other hand, as the drug concentration crosses a threshold, this results in the eventual triggering of cell death throughout the domain. Simulations with even higher levels of input reveal tumour cells being destroyed faster. These results are easy to rationalize since if the threshold level of drug signal required to trigger apoptosis is not crossed, no drug-induced death will occur. On the other hand, when the threshold is crossed, all cells will die, due to the fact that drug is infused at a constant rate and also because no element of variability or stochasticity is assumed in the model. This trend, seen for both the signalling variants, is intuitive, noting that for constant levels of stimuli, the signalling behaves like simple switches with threshold effects.

**Figure 3:**
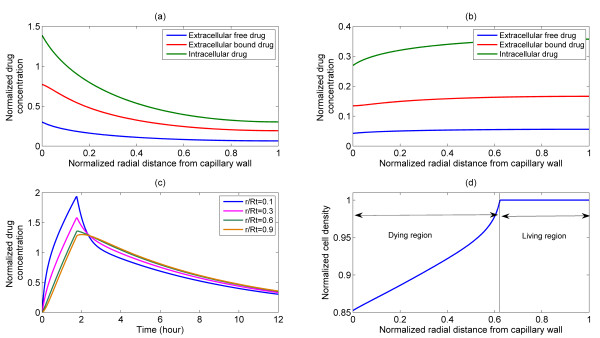
**Representative plots of spatial and temporal distributions of drug and cell density following a pulse injection for bistable switch**. Upper panel: Intracellular and extracellular drug distribution in the tumour cord under pulse injection, S = 4, T = 1.75 h, profiles shown at (a) 30 min, (b) 12 h. Bottom left: (c) time course of intracellular drug distribution at various radial positions. Bottom right: (d) spatial profile of cell density at 12 h, revealing cell death and living regions in the front and rear of the tumour cord.

The case of a bolus (pulse) injection, which is a more realistic form of injection is considered in Figure 3. Following a pulse injection, the extracellular drug concentration increases everywhere in the interstitium. In the early stages the extracellular concentration has a decaying spatial profile, but as time progresses, it starts to flatten out while revealing a small spatial variation. The results also demonstrate how, as the injection period is passed, the extracellular concentration starts to decline (Figure 3(c)). In the transient period of elevated extracellular drug concentration, the drug is pumped into the cells and is able to activate the intracellular pathways, and eventually induce apoptosis. This latter aspect is clearly seen in Figure 3(d) where the tumour cell density in the region near the wall starts decreasing and eventually becomes zero. However, there is a farther region where no cell death occurs, suggesting that a bolus is able to induce cell apoptosis in a well defined spatial region. The simulation results shown in Figure 3 were performed with the bistable switch.

**Figure 4:**
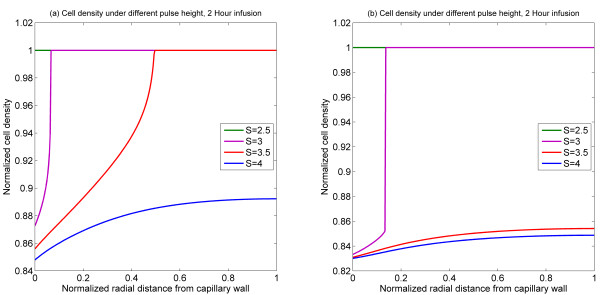
**Spatial profiles of cell density at 12 h for 2 h injection of different pulse heights**. (a) Bistable switch case, (b) Monostable switch case.

In order to examine the effects of the pulse strength (for fixed time) and the differences between monostable and bistable cases, a series of simulations were performed (Figure 4). The simulation results in Figure 4(a), representing a temporal snapshot of the cell density at 12 hours (for a 2 hour bolus injection), reveal that depending on the strength of the pulse injection the following trend is observed. For relatively weak strengths, no effect of the drug on the tumour cell density profile is seen. As the strength of the injection is increased, cell density begins to decline in a region adjacent to the capillary wall. Further increasing the strength of the injection leads to a broadening of the region where cell death occurs, and eventually as the pulse strength becomes high enough, cell apoptosis can be triggered throughout the domain. This is because, even though the activating signal is transient, it can trigger an irreversible change by its interaction with a bistable switch. The qualitative aspects of such interaction are discussed in Seaton *et al *[18]. Figure 4(b) shows the corresponding results with the monostable switch. Here again, the same basic trend can be observed, except that there is a relatively narrow region in parameter space which leads to partial cell death. This in turn can be explained by the fact that owing to the intrinsic dynamics the bistable switch is a more sensitive differentiator between transient signals than a monostable switch. Another point worth noting is the fact that in both the monostable and bistable cases, the width of the cell death zone is a strongly non-linear function of the injection level.

**Figure 5:**
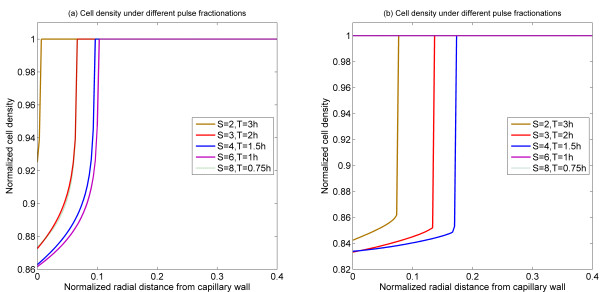
**Spatial profiles of cell density at 12 h for different fractionations**. (a) Bistable switch case, (b) Monostable switch case. As the pulse injection time is reduced, the region of killing shrinks in both cases, and in fact killing is eliminated in the monostable case.

Numerical simulations were also performed for different combinations of pulse height and infusion time, while keeping their product (i.e. total amount of drug) fixed. Results shown in Figure 5 suggest that when the infusion time is too short or too long, little cell death occurs. There is an intermediate infusion time at which cell death reaches the highest level. Again similar trends are observed in the monostable and bistable cases, except that a broader spectrum of the cell death zone is observed in the monostable case. These results may be explained as follows. For a fixed amount of drug, if the infusion time is high, then the pulse height becomes low and no cell apoptosis is triggered, noting that even in a constant step input, no cell death is found for a sufficiently low level of infusion (Figure 2). On the other hand, if the infusion time is too short, there would be very little time for the drug to enter into the interstitium and act on cells to cause cell death. Hence an optimal infusion time for causing maximum cell death will likely exist. Of course, it is recognized that such an analysis is based on the asymptotic limits, and that arbitrarily high signal levels may not be possible in practice (either due to injection, or transcapilliary transport limitations).

**Figure 6:**
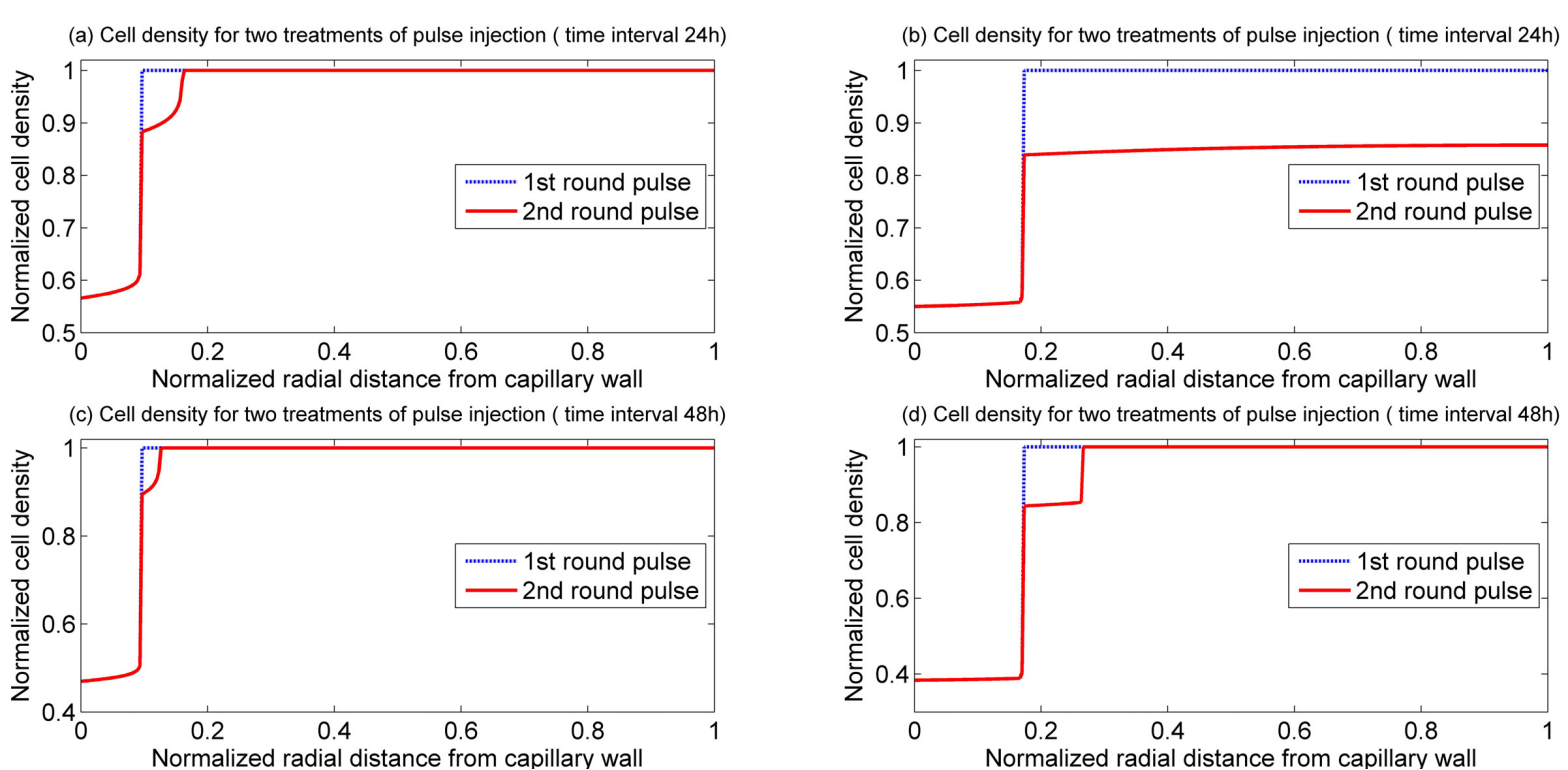
**Spatial profiles of cell density for two rounds of pulse injection with the same pulse height and weight, S = 4, T = 1.5 h**. Upper panel: cell density at 36 h with 24 h interval between pulses, (a) Bistable switch case, (b) Monostable switch case. Lower panel: cell density at 60 h with 48 h interval between pulses, (c) Bistable switch case, (d) Monostable switch case.

It is also interesting to examine the effects of double bolus injections. Figure 6 shows the response of the tumour to two bolus injections in both monostable and bistable cases. Two different values of the time interval between the first and second bolus injections are tested. It can be observed that a second bolus injection does indeed increase the width of the cell death zone, and that an increase in the time interval between injections has a detrimental effect on cell killing. Thus in the bistable case, doubling the time interval causes a substantial reduction in the effect of the second bolus. In the monostable case, for the parameters chosen, a second bolus could cause cell death in the entire domain while the first bolus did not. However, even here increasing the time interval between injections reduces the effectiveness. This is because the second bolus is unable to make use of the residual effect from the first bolus. It should be mentioned that while a second bolus expands the cell death zone, it has a very weak effect in accelerating cell death in regions which are already part of the death zone. The results of this simulation also indicate that one should be careful about attributing such effects to drug resistance, since these trends are observed even without any resistance.

**Figure 7:**
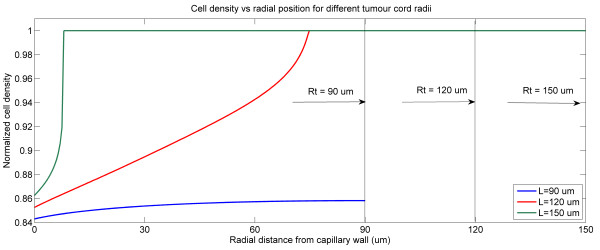
**Spatial profiles of cell density at 12 h for different tumour cord radii under a pulse injection, S = 4, T = 1.75 h for bistable switch**.

As part of the analysis, the effects of other model parameters are also examined. Generally, varying the threshold for cell apoptosis and intracellular kinetic parameters has the expected effect with regard to destroying cells. Shown in Figure 7 are the effects of the size of the tumour cord on the response of tumour to a bolus injection as considered in Figure 3. The results show that as the tumour cord radius is increased, the effect is to shrink the cell death region, in absolute terms. This is because the drug is now spread out over a large region and becomes diluted. On the other hand a constant infusion would result in the same effect independent of the tumour cord radius (though the time for causing cell death may vary).

## Discussion

In this paper we have taken the first steps towards a comprehensive systems-framework to investigate the effect of drugs on solid tumours. Our ultimate goal is to combine different aspects of the fluid mechanics and drug transport as well as intracellular processes to develop a comprehensive framework for elucidating the role of different factors which affect the efficacy of anti-cancer drugs. A particular impetus comes from the area of systems biology, where detailed investigations of intracellular processes have been performed in the past decade, with the promise that the fruits of such investigations will have an impact on human health and disease control.

Our approach is motivated by the desire to create an appropriate framework which is based on a dynamical systems underpinning, which can seriously tackle different aspects of the cellular complexity, and the non-linearity of cellular signal processing. We recognize that for this a predictive systems framework is needed.

Thus our immediate focus is not to fit data (see comments below).

The systems framework proposed in this study incorporates three different levels of description in a coupled manner: the dynamics of the extracellular drug, which includes details of the drug transport; the intracellular dynamics; and the population dynamics of tumour cells. Our framework incorporates basic descriptions of what we regard as the minimal elements to obtain a mechanistic description connecting drug input to cell killing, dynamically. For simplicity, a basic cylindrical-shaped tumour-cord model is analysed which contains a capillary at the centre and an interstitium in the surrounding annular space. Different modes of drug injection are simulated and model parameters corresponding to doxorubicin are used. As a first step, blood flow in the capillary vessel is not modelled explicitly (but will be included at the next stage), instead an effective drug concentration at the capillary wall is specified to represent the mode of drug injection. The extracellular drug transport in the interstitium primarily includes drug diffusion, uptake by the cell and pumping out from the cell. The latter elements depend on the local cell density. The intracellular description also incorporates the pumping in and out of drug, and minimal descriptions of the drug-triggered cell death. In this case rather than accounting for all the biochemical details, many of which are still unknown, coarse grained descriptions of signalling pathways are employed, which account for qualitatively different characteristics of the signal transduction dynamics (one involving a monostable irreversible switch, and the other a bistable switch). Our intracellular descriptions compactly encapsulate the qualitative nature of information flow in the cell.

The population level description of cells uses a basic logistic model which captures the cell birth and death in a continuum description. Since the present model only provides a skeletal description of the processes involved, the focus here is to obtain predictions regarding qualitative trends rather than quantitative values. At the same time this is very important, in order to seriously represent appropriately the complexity from the cellular side in a manner which can be systematically augmented. Overall, our modelling takes a hybrid approach which seeks to retain the predictive advantages of mechanistic models, while coarse graining intracellular descriptions to retain the relevant input-output signalling characteristics. We also avoid casting the intracellular models in concrete biochemical terms, but will do this subsequently, using this framework as a skeleton.

Two variants of intracellular description are examined to address the important question as to whether the difference between these descriptions plays an important role in the current context. Both models (monostable and bistable) are used to analyse different forms of drug input, such as sustained drug input, single and multiple bolus injections. The numerical results demonstrate that sustained drug injection results in either the entire tumour tissue being destroyed or the entire tissue surviving, depending on the concentration of drug. In the case of a single bolus, cell death occurs in a region close to the capillary wall, and the size of this region increases with an increase in the time period or concentration of the bolus. This also means that subsequent bolus injections will result in deeper penetration of the drug and hence broadening the region where cells are destroyed. Interestingly, the essential trends predicted by the monostable and bistable switches are similar, suggesting that the complexity of cellular signal processing notwithstanding, the responses to stimuli of the kind which may be encountered in realistic drug treatment are in fact basically similar. Differences could arise when one considers more complex temporal stimuli. We extended our analysis to a case where drug is released at a location away from the tumour and the drug concentration input to the tumour is determined using a pharmacokinetic model [19]. Results (not shown here) show a much smaller cell death zone, since a fair portion of the drug has been absorbed in other parts of the body. All these demonstrate that our model can be used to systematically investigate the cellular response to different forms of drug injections.

It is clear that the proposed modelling framework is the first step and thus has a number of limitations. Firstly, the most basic descriptions are used for the three levels of the modelling. Further work to include more biochemical details of the signalling network (including the detailed dynamics of the caspase network and its regulation) is needed in order to obtain a more detailed depiction of the cellular signalling. Clearly additional elements may involve either feed forward pathways, or possibly even multiple-switch type signalling, as well as redundant pathways, which may have a quantitative effect on the dynamics of the tumour. However since the two signalling models examined here, which are qualitatively completely different, produce very similar basic trends, we expect that essential conclusions arising purely from these additional variants in the signalling are likely to play a minor role. Indeed basic simulations of both the models we employ and a more detailed model of Tyson and co-workers [14] at the ODE level reveal similar characteristics of response for the types of signals considered. Therefore, the general response of more complex models is expected to indicate essentially similar trends. Based on our analysis it can be speculated that the additional layers of internal feedback/complexity are likely to be more important in affecting the tumour response to multiple bolus stimuli which are not too spaced out. It is worth pointing out that if signalling includes both a bistable switch and a downstream irreversible element (combining features of our models, with the switch effect being realized by a bistable circuit, but the irreversibility present through a downstream irreversible switch), we expect the behaviour to be very similar to the monostable switch we examined, for simpler temporal signals; in more complex temporal signals, we expect the bistable feature of signalling to play a dominant role in the signal transduction and response (analyzed subsequently). A related point to be made is that one should be very careful about claims of validating models which include cellular signalling, from scanty data.

Secondly, the present model does not include any cell variability, heterogeneity or cellular interaction. We fully recognize that inclusion of such factors can lead to partial survival of cells. Although incorporation of these factors may allow some fraction of cells to survive in a particular region, they need to be examined systematically to understand both their individual and combined effects. Noting that the complexity and specifically the non-linearity of cellular signal transduction will play an important role in conjunction with these, the entire modelling of these factors must be examined in a systematic and thorough manner, and this will be done carefully building on this framework. A similar comment can be made on cell cycle effects (which is of special relevance to analyzing the effect of drugs which target different stages of the cell cycle). Likewise the effects of factors which confer cell resistance need to be examined. All the above mentioned factors involve additional aspects of the cellular description and response. Other elements include the description of cell movement, and the effect of realistic microvascular geometries with comprehensive fluid mechanical descriptions.

Noting that multiple combinations of these effects may be present in different tumour types, it is vital to assess the roles of these factors in a systematic and predictive way, which makes clear testable predictions, and where possible to assess the role of these factors individually and together in an unambiguous fashion. This underscores our approach in dealing with the complexity of this system.

## Conclusions

Overall we have combined three levels of description to take the first steps towards a comprehensive systems description of drug effects on tumour cells. Since a particular focus in the future is to use this platform to investigate different effects of cellular complexity, a basic model structure has been adopted in the first stage. This is vital in order to build a credible platform which seriously and systematically addresses different aspects of cellular complexity. This in turn would be a necessary bridge to effectively exploit the progress in systems biology of cellular processes and bring this to bear on the problem of understanding drug delivery processes and improving their efficacy in eliminating tumour cells.

## Competing interests

The authors declare that they have no competing interests.

## Authors' contributions

CL, JK and XYX jointly planned the work, CL contributed the computational results presented here, and JK wrote the paper, with CL and XYX. All authors read and approved the final manuscript.

## Appendix

In this section, we briefly discuss some features of the model equations which are relevant to the work presented here. While we will not perform a detailed and exhaustive theoretical analysis, we examine some selected aspects which complement the numerical work.

We first start by examining the steady state of the kinetic monostable and bistable modules. In the case of the monostable threshold module, the steady state of the module is given by

(A1) $$R=I^{n}∕\left(k_{h}+I^{n}\right)$$

(A2) $$R_{1}=\frac{R}{R+k_{r}∕k_{f}}$$

where I is the upstream signal (in our case the intracellular drug concentration). Since the first equation is a Hill type equation, we see that if I is above the threshold, this results in a steady state of R which is essentially 1, and further a high level of R_1 _which triggers the threshold. Note that in this model, once the threshold for apoptosis is triggered, the irreversibility implies that subsequent dynamics or approaching of a steady state for the monostable module variables R and R_1 _are irrelevant. Thus it is important to also look at the dynamics. In general, for a time varying signal I(t), we have

(A3) $$R\left(t\right)=R\left(0\right)exp\left(-kt\right)+exp\left(-kt\right)\int_{0}^{t}\left(\frac{kI{\left(w\right)}^{n}}{k_{h}+I{\left(w\right)}^{n}}\right)dw$$

from which R_1_(t) can be calculated. Now the important piece of information from the dynamic variation of R_1 _is whether or not the threshold value is crossed for a given static or time-varying input I, and at what time the threshold is crossed.

**Figure 8:**
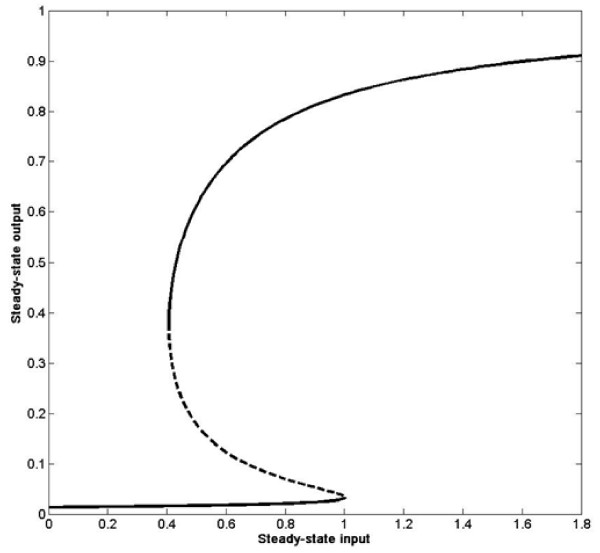
**Steady state input-output curves**.

The bistable model is one which involves positive feedback. In this case, the steady state of R in terms of the input I is a cubic equation, which is difficult to solve analytically. However, using numerical bifurcation analysis, one can determine the steady state of R as a function of I. This is shown in Figure 8. We see that at the reference basal condition, the system has two stable steady states (and an intermediate unstable steady state). The important point to note is that as I crosses its threshold of 1, the system moves into the monostable regime and evolves to a higher steady state level. However if I subsequently decreases below the threshold, the system may or may not come back to the lower steady state. Depending on the time spent beyond the threshold, the system may either return to its original state or be permanently switched on, indicating an irreversible transition.

As mentioned in the text, when R_1 _crosses a threshold, a significant lowering of the growth rate in the population description is implemented. This is done explicitly in an irreversible way in the monostable model simulations, and in a reversible way in the bistable model simulations. Clearly from the discussion above, we see that both models embody the fact that if the signal is above a threshold level for a sufficient period of time, this can lead to cell killing.

We now discuss some aspects of the distributed model. Looking at the logistic model for the growth rate, we see that the basal steady state is c_t _= a/b, where a = a_1 _**- **a_2 _with a_1 _and a_2 _being the linear growth and death rates, with a_1 _**>**a_2_. When the apoptosis threshold is crossed in the model, a_1 _is reduced to a value less than a_2_. Thus as a result of the cellular dynamics, in both the monostable and bistable cases, a second steady state exists which is c_t _**= **0, which is the attained state if the threshold is crossed (note that in our monostable model if the threshold is temporally attained, then this is the only eventual state which can be reached, while in the bistable case one can say that if the threshold remains crossed at steady state, the cell density eventually becomes 0). Thus it is possible at steady state at any location for the steady state to be the basal level or to become 0. Our simulations provide ample evidence of the fact that some region gets killed completely, while others remain/regain their basal level.

We can also comment on the steady state levels of the extracellular drug concentration. An analysis of the equation for the intracellular drug concentration reveals that at steady state there is a balance between the pumping in and out of the drug. Likewise, by examining the binding to and unbinding from albumin in the interstitium, and considering the steady state of the bound extracellular drug (in the particular case where the bound drug is regarded as non-diffusible), we immediately see that this binding unbinding reaction must be at equilibrium. Thus at steady state, the extracellular drug concentration satisfies

(A4) $$k_{d}\nabla^{2}E=0$$

which implies that the extracellular drug concentration is spatially uniform and this depends on the level of drug being pumped in. If a persistent stimulus is maintained, the drug concentration in the interstitium attains a commensurate value. If the stimulus was applied as a bolus, it means that eventually no drug is pumped in and at steady state the extracellular drug concentration is zero everywhere. However there can be regions in the interstitium where the cell density is zero, as we have seen in the simulations and from the discussion above. Thus at steady state the extracellular drug concentration will be uniform but the tumour cell density can be non-uniform.

The dynamics of the coupled system may also be studied. Without presenting this in greater detail here, we comment that the variation of the cell density is much slower than the remaining dynamics, so that the remaining dynamics can be analyzed using a perturbation analysis where the cell density variation occurs on a slower time scale.

### Supplementary results

In this subsection, we present some additional results to supplement those presented in the main text. We briefly present two sets of results, both related to bolus injections.

In the case of the single bolus injection, the effect of a single bolus on cell killing was examined, and the effect of bolus fractionation was considered as well. Here we present results related to the effect of infusion time of a bolus injection.

**Figure 9:**
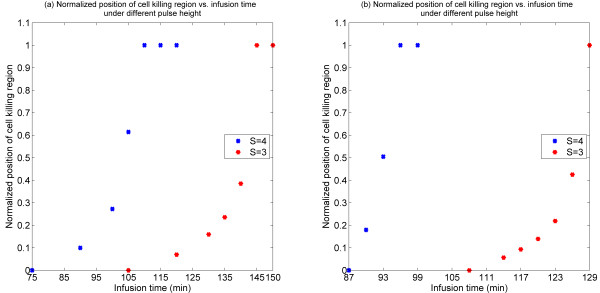
**Normalized position of cell killing region as a function of infusion time for different pulse heights**. (a) Bistable switch case, (b) Monostable switch case.

The effect of infusion time of bolus injection is shown in Figure 9 where it is seen that an increase in infusion time broadens the cell death region and does so in a very non-linear fashion. Again the same trends are observed in both monostable and bistable cases.

**Figure 10:**
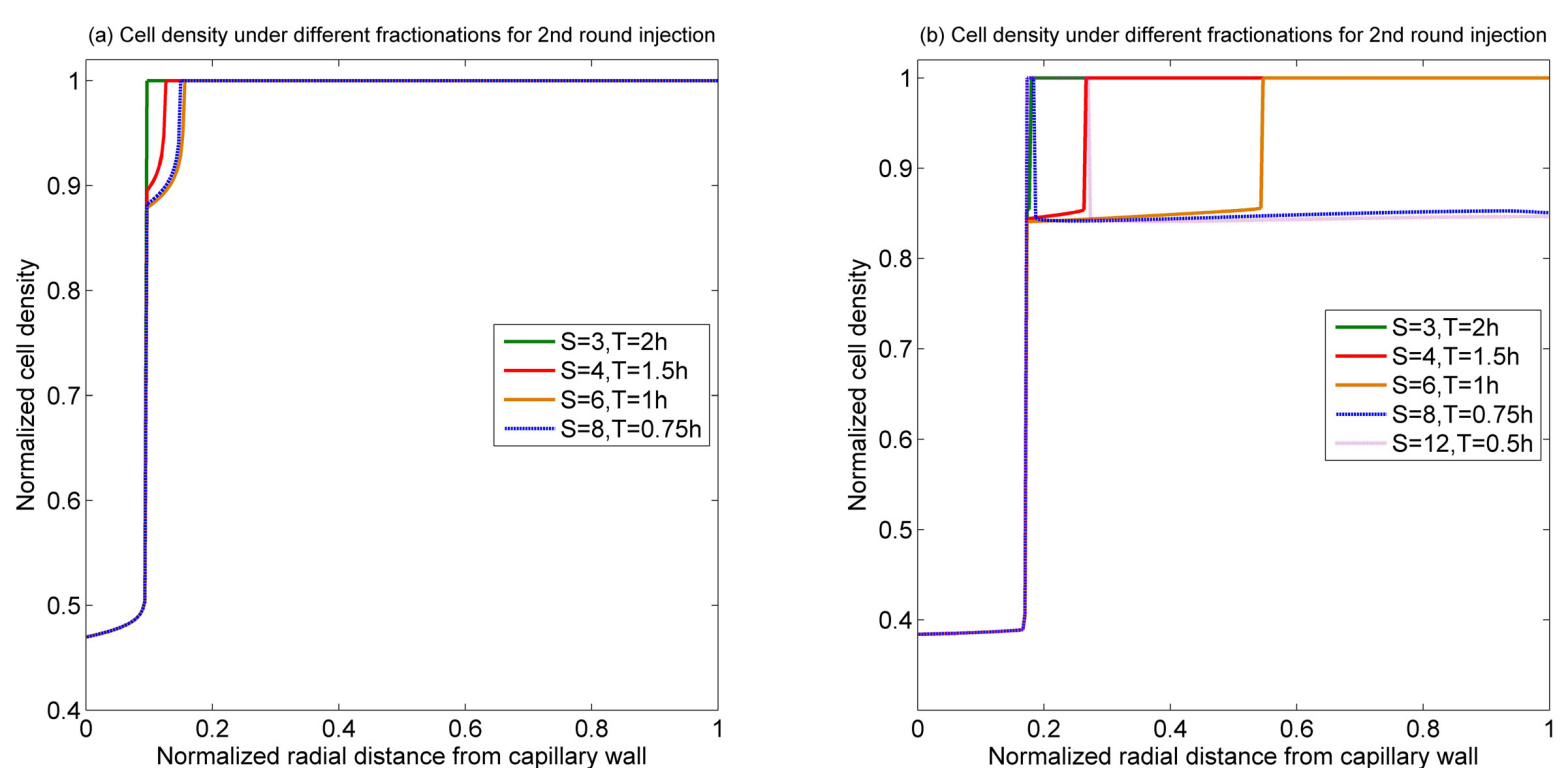
**Spatial profiles of cell density at 60 h for the 2^nd ^injection under different fractionations with 48 h time interval**. (a) Bistable switch case, (b) Monostable switch case.

The effect of double bolus injections are considered in the text. To extend these results, we briefly examine how the second bolus may be fractionated for a fixed first bolus. The effect of second bolus fractionation is shown in Figure 10. For a fixed first bolus and time interval between injections, there seems to be an optimal way of fractionating the second bolus. It is worth pointing out that in the monostable case, the possibility of killing regions separated by a living region is seen.
